# Baja captación y éxito en el tratamiento para la tuberculosis en una cárcel de Ecuador

**DOI:** 10.26633/RPSP.2019.106

**Published:** 2019-12-30

**Authors:** Félix Chong, Diana Marín, Freddy Pérez

**Affiliations:** 1 Ministerio de Salud Pública del Ecuador Ministerio de Salud Pública del Ecuador Ecuador Ministerio de Salud Pública del Ecuador, Ecuador.; 2 Universidad Pontificia Bolivariana Universidad Pontificia Bolivariana Colombia Universidad Pontificia Bolivariana, Colombia.; 3 Departamento de Enfermedades Transmisibles Determinantes Ambientales de la Salud Organización Panamericana de la Salud Washington D.C. Estados Unidos de América Departamento de Enfermedades Transmisibles y Determinantes Ambientales de la Salud, Organización Panamericana de la Salud, Washington D.C., Estados Unidos de América.

**Keywords:** Tuberculosis, prisioneros, Ecuador, Tuberculosis, prisioners, Ecuador, Tuberculose, prisioneiros, Ecuador

## Abstract

**Objetivo.:**

Evaluar el control de la tuberculosis pulmonar en un centro de privación de la libertad e identificar los factores de riesgo asociados con tratamiento no exitoso en la cárcel más grande en Ecuador.

**Métodos.:**

Se analizaron los datos de vigilancia de la prisión y de una cohorte de internos diagnosticados con tuberculosis (TB) entre los años 2015 y 2016. Se excluyeron los registros sin desenlace en el tratamiento. Se estimó el porcentaje de sintomáticos respiratorios (SR) identificados y la tasa de incidencia de TB. Los factores asociados con el tratamiento no exitoso se estimaron con regresión logística binomial.

**Resultados.:**

De 59 846 consultas médicas, 3% se identificó como SR y, de estos, 326 reclusos tenían TB, 184 fueron analizados. La tasa de incidencia de TB en la prisión fue de 3 947/100 000 habitantes. El porcentaje de tratamiento exitoso fue de 70,4% (65,6% curado y 4,8% con tratamiento completo) y 29,4% de tratamiento no exitoso (12,5% de pérdidas durante el seguimiento, 5% fallecieron, 1,1% de fracasos de tratamiento y 10,8% no fueron evaluados). La seropositividad para el virus de la inmunodeficiencia humana (VIH) se asoció con un mayor riesgo de tratamiento no exitoso (riesgo relativo: 1,66, intervalo de confianza de 95%: 1,33-2,07).

**Conclusión.:**

La incidencia de TB en la prisión es 123 veces más alta que en la población general de Ecuador. Los prisioneros coinfectados con TB-VIH tienen mayor riesgo de no tener un tratamiento exitoso y se requiere articulación entre los ministerios de salud y de justicia que permita la implementación adecuada de protocolos de salud y de la *estrategia Fin a la TB.*

## Abstract (en)

**Objective.:**

Evaluate the control of pulmonary tuberculosis in a detention center and identify the risk factors associated with unsuccessful treatment in the largest prison in Ecuador.

**Methods.:**

Surveillance data from the prison and a cohort of inmates diagnosed with tuberculosis (TB) between 2015 and 2016 were analyzed. Records without treatment outcome information were excluded. The percentage of patients with respiratory symptoms and TB incidence rate were estimated. Factors associated with unsuccessful treatment were estimated with binomial logistic regression.

**Results.:**

Of 59 846 medical consultations, 3% of respiratory symptoms were identified and, of these, 326 inmates had TB; 184 of them were analyzed. The incidence rate of TB in the prison was 3 947/100 000 inhabitants. Treatment was successful in 70.4% (65.6% cured; 4.8% treatment completed) and unsuccessful in 29.4% (12.5% lost during follow-up, 5% deceased, 1.1% treatment failure, 10.8% not evaluated). Seropositivity for human immunodeficiency virus (HIV) was associated with an increased risk of unsuccessful treatment (relative risk: 1.66, 95% confidence interval: 1.33-2.07).

**Conclusion.:**

The incidence of TB in the prison was 123 times higher than in the general population of Ecuador. Prisoners co-infected with HIV-TB are at greater risk of not having a successful treatment, and articulation is required between the ministries of health and justice that allows the proper implementation of health protocols and the End TB Strategy.

## RESUMO

**Objetivo.:**

Avaliar o controle da tuberculose (TB) pulmonar e identificar os fatores de risco associados ao tratamento malsucedido na maior prisão do Equador.

**Métodos.:**

Foram analisados os dados de vigilância da prisão e de uma coorte de reclusos da mesma prisão, diagnosticados com TB em 2015 e 2016. Foram excluídos os registros sem resultado do tratamento. A porcentagem de sintomas respiratórios identificados e a taxa de incidência de TB foram estimadas. Os fatores associados ao tratamento malsucedido foram estimados com regressão logística binomial.

**Resultados.:**

Em 59 846 consultas médicas, 3% dos reclusos foram identificadas com sintomas respiratórios. Entre esses, 326 tinham TB e 184 foram analisados. A taxa de incidência de TB na prisão foi de 3 947/100 000 habitantes. Observou-se tratamento bem-sucedido em 70,4% (65,6% curados e 4,8% com tratamento completo) e malsucedido em 29,4% (12,5% perdidos durante o acompanhamento, 5% morreram, 1,1% com falhas do tratamento e 10,8% sem avaliação). A soropositividade para HIV foi associada a risco aumentado de tratamento malsucedido (risco relativo 1,66; intervalo de confiança de 95%: 1,33 a 2,07).

**Conclusão:**

A incidência de TB na prisão foi 123 vezes maior do que na população geral do Equador. Prisioneiros coinfectados com HIV-TB correm maior risco de não ter tratamento bem-sucedido. É necessária uma articulação entre os ministérios da saúde e da justiça para implementar de forma adequada os protocolos de saúde e da Estratégia pelo Fim da TB.

En el año 2018, Ecuador ocupó el décimo puesto de los países con carga más alta de tuberculosis (TB) en América Latina y el Caribe, con una tasa de incidencia de 44 casos notificados por cada 100 000 habitantes (1, 2). En el país no existe información precisa con respecto a los casos notificados por año en personas privadas de libertad afectadas de TB.

La TB en los centros de privación de libertad (CPL) es considerada un tema prioritario a nivel mundial, debido a la alta tasa de incidencia de esta patología que alcanza a ser de cinco a 50 veces mayor que en la población general (3-5). Los CPL son considerados posibles fuentes de reservorio por su déficit en las medidas de control de infecciones de tuberculosis, hacinamiento, demoras en el diagnóstico y falencias en la atención directa a la persona privada de libertad (PPL), lo que los convierte en un grupo vulnerable (6, 7). Este grupo de población presenta, a su vez, enfermedades asociadas como desnutrición, farmacodependencia, infección por virus de la inmunodeficiencia humana (VIH) y diabetes. Estas son algunas de las causas por las cuales el tratamiento para la tuberculosis no es exitoso en este grupo de afectados (8). Esto trae como consecuencia la diseminación de la enfermedad en la comunidad y el incremento de los costos en el tratamiento para el Estado (9-12).

En el Ecuador, provincia del Guayas, se encuentra la Zona 8-Salud, la cual cuenta con una población total de 3 029 320 habitantes y está conformada por 12 distritos con sus respectivos establecimientos de salud. El distrito de salud 09D07 tiene una población total de 286 246 habitantes y cuenta con siete establecimientos de salud y dos centros de privación de libertad (CPL-Regional Zona 8 y CPL-Guayas 1 varones). El estudio se realizó en el CPL-Guayas 1 (G1) varones caracterizado por un alto índice de hacinamiento y falta de áreas de separación para los pacientes con diagnóstico de TB. La Estrategia de Prevención y Control de Tuberculosis en el CPL-G1 varones se implementó hace aproximadamente 10 años, y ha venido realizando las actividades de captación activa de sintomáticos respiratorios, con apoyo para el diagnóstico de la red de laboratorios del Ministerio de Salud Pública mediante la remisión de la muestra al laboratorio del distrito al que pertenecen y al hospital neumológico de referencia “Alfredo J. Valenzuela”. Luego de obtener el resultado, el personal de salud del centro de privación suministra el tratamiento directamente observado (TDO) y se encarga del seguimiento de los pacientes que deben ser notificados al sistema de información de TB. A todos los pacientes con diagnósticos de TB por baciloscopia positiva (BK+) se les realiza tamizaje para VIH y diabetes; de presentar cualquiera de las dos patologías se efectúa la atención y el seguimiento médico respectivo de acuerdo con las normas nacionales (13, 14).

La Zona 8 (Guayaquil, Durán, Samborondón) tiene una tasa de incidencia de TB de 91/100 000 habitantes, y representa tres veces más la incidencia de TB del país; en esta zona, el CPL-G1 varones cuenta con 8 700 PPL; su capacidad total aproximada de internamiento es de 4 400 PPL. Los factores asociados a un tratamiento no exitoso de la TB no están bien documentados en los CPL de Ecuador. Esto y la falta de información confiable sobre la situación de la enfermedad por parte de las autoridades locales y nacionales hacen imposible trazar objetivos para controlar la TB y estrategias para lograrlos. En este contexto, los objetivos del estudio fueron evaluar el control de la tuberculosis pulmonar en el CPL-G1 varones e identificar los factores de riesgo asociados con el tratamiento no exitoso de la TB.

## MATERIALES Y MÉTODOS

Se realizó una investigación operativa que incluyó un diseño transversal descriptivo para evaluar el control de la tuberculosis pulmonar dentro del CPL y un diseño de cohorte retrospectivo para determinar los factores asociados con el tratamiento no exitoso.

Para la evaluación del control de la tuberculosis se utilizaron las estadísticas de la población y las consultas en mayores de 15 años suministradas por la Dirección del CPL y los informes trimestrales, anuales y bases de datos de la Estrategia de Prevención y Control de Tuberculosis del CPL-G1 varones. Se calcularon los indicadores programáticos de detección de sintomáticos respiratorios, de diagnóstico de tuberculosis y detección de VIH (13). El porcentaje de SR identificados entre las consultas en mayores de 15 años se comparó con la meta de 4% establecida por el Ministerio de Salud Pública del Ecuador (13).

Para el análisis de los factores asociados con tratamiento no exitoso se estudiaron las personas privadas de libertad (PPL) afectadas con tuberculosis durante el período enero de 2 015 a diciembre 2 016 que recibieron tratamiento, se encontraban registradas en las bases de datos de la estrategia de tuberculosis del CPL-G1 varones y contaban con la información registrada en las tarjetas de registro del tratamiento antituberculoso.

Se analizaron las variables de edad en años, peso corporal en kilogramos, zona de origen, forma clínica (pulmonar, extrapulmonar), condición de ingreso (nuevo, recaída, pérdida en el seguimiento), frotis y cultivo de diagnóstico (negativo, +, ++, +++), prueba de tamizaje para VIH (reactivo, no reactivo, sin prueba), comorbilidad con diabetes (sí, no) y tiempo en días de inicio de tratamiento para tuberculosis (3 o menos, más de 3). Para el desenlace del tratamiento (curado, tratamiento terminado, pérdida en el seguimiento, fracaso o fallecido), control bacteriológico (frotis, cultivo y PCR en tiempo real) y demás definiciones operacionales, se utilizaron las definiciones de la Organización Mundial de la Salud (OMS) (15).

**FIGURA 1.**
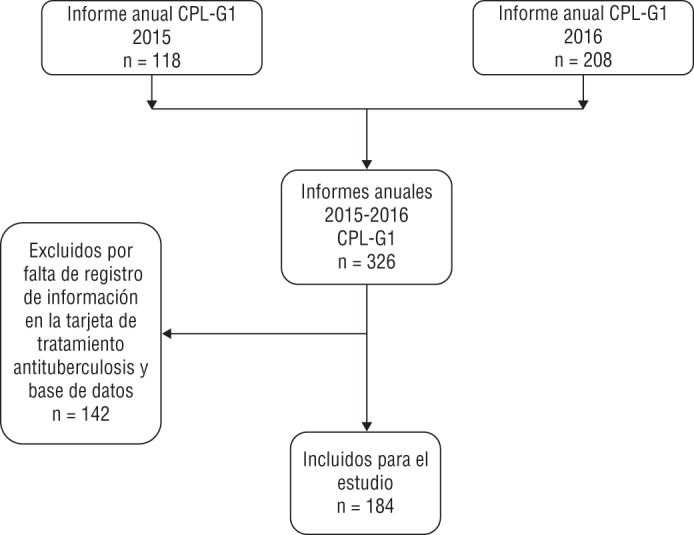
Flujograma de personas privadas de libertad con diagnóstico de tuberculosis en el CPL-G1 varones, analizadas de los informes anuales de la Estrategia de Tuberculosis, Ecuador (2015-2016)

Elaboración propia, con base en datos obtenidos de los informes anuales de la Estrategia de Prevención y Control de Tuberculosis.

**CUADRO 1.** Indicadores epidemiológicos y operacionales de tuberculosis en el centro de privación de la libertad Guayas 1 varones (CPL-G1), Ecuador (2015-2016)

Indicador	n	%
Detección de sintomáticos respiratorios (SR)^a^			
Consultas en mayores de 15 años	59 846	100
SR esperados	2 394	4
SR identificados	1 547	3
SR identificados/SR examinados	1 547/1 547	
Casos según la forma de tuberculosis (TB)			
Total de casos de TB en todas sus formas	326	21
Casos nuevos	307	20
Casos previamente tratados	19	1
Casos de TB con frotis positivo^b^	267	87
Casos de TB con frotis negativo y cultivo positivo^b^	38	12
Cassos de TB extrapulmonar^b^	2	0,7
Detección de VIH			
Casos de coinfección TB/VIH^c^	18	6
Tasa de incidencia de TB^d^		
En CPL-G1 varones	3 947	
En Zona 8^e^	80,42	
En el país	31,88	

a Sintomático respiratorio: persona que tiene tos con flema durante más de 15 días (27).

b Entre el total de casos nuevos.

c Entre el total de casos de TB de todas las formas.

d Por cada 100 000 habitantes.

e Zona 8: ciudad de Guayaquil, Cantón Durán y Samborondón.

TB, tuberculosis; VIH, virus de la inmunodeficiencia humana.

Cuadro de elaboración propia con base en datos obtenidos de los informes anuales de la Estretegia de Prevención y Control de Tuberculosis.

Para responder a los diferentes objetivos del estudio se contó con el número de personas privadas de libertad diagnosticadas de tuberculosis por año y se estimó la incidencia anual con su intervalo de confianza de 95% (IC95%). Para describir las características y evaluar el seguimiento al tratamiento se tomaron las frecuencias absolutas y porcentajes y para variables cuantitativas como la edad, se estimó la mediana y rango intercuartílico (RIC: Q1-Q3).

Para establecer los factores que se asociaron con el tratamiento no exitoso (pérdida en el seguimiento, fracaso, fallecido) (15) se utilizó la prueba de chi cuadrado y se calculó el riesgo relativo (RR) crudo de un tratamiento no exitoso IC95%. Además, en la literatura, las variables han demostrado estar asociadas con bajas tasas de éxito fueron incluidas en la regresión y se reportó el RR ajustado con un modelo de regresión log-binomial, se utilizó un nivel de significación bilateral de 0,05.

En el análisis multivariado se excluyeron aquellas PPL cuya condición de egreso fue no evaluado (transferencia sin confirmar). Los análisis se realizaron con los programas Epi Info 7.0^®^ y Stata 15.0^®^.

Se contó con la autorización del Comité de Ética del Hospital de Infectología “Dr. José Daniel Rodríguez Maridueña” de la provincia de Guayas en Ecuador y del Comité de Revisión de Ética de la Organización Panamericana de la Salud (PAHOERC). La investigación se clasificó sin riesgo para los participantes debido a que la población de estudio ya había culminado el tratamiento para la tuberculosis y, por lo tanto, no era posible tener acceso directo a ellos. Los datos para el primer objetivo eran agregados y, para el segundo, por el tipo de población se solicitó al PNCT que entregaran la base de datos sin número de identificación, nombres ni dirección de residencia para garantizar el anonimato de los participantes.

## RESULTADOS

Los informes anuales de los años 2 015 y 2 016 demuestran un total de 59 846 consultas en los mayores de 15 años donde se identificaron 1 547 (3%) SR, de los cuales 326 PPL presentaron TB en todas sus formas (figura 1). De los 326 PPL afectados con TB, 307 (94%) fueron casos nuevos. El CPL-G1 varones muestra una tasa de incidencia de 3 947 por 100 000 habitantes, la Zona 8 de 80,42 por 100 000 y la de nivel país de 31 por 100 000 (cuadro 1).

**FIGURA 2.**
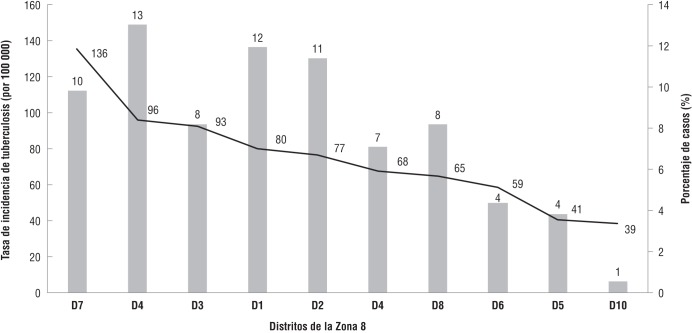
Porcentaje de casos de tuberculosis y tasa de incidencia en los distritos de salud de la Zona 8, Ecuador (2015-2016)

**FIGURA 3.**
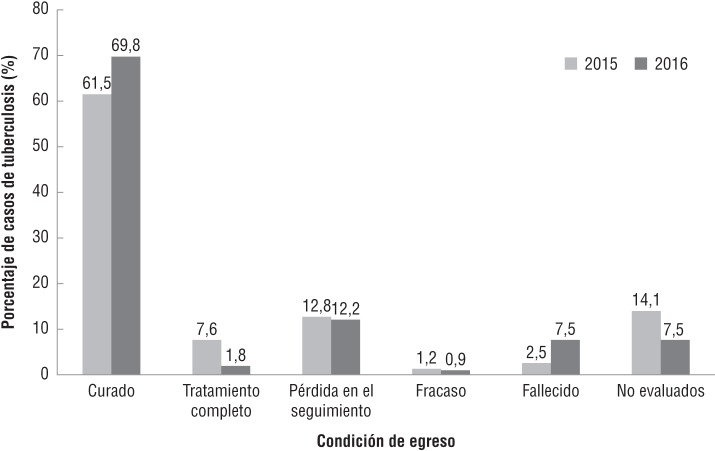
Seguimiento y condición de egreso de las personas privadas de libertad afectadas con tuberculosis del CPL-G1 varones, Ecuador (2015-2016)

De los 326 pacientes diagnosticados y notificados en la base de datos de la estrategia de tuberculosis del CPL, se excluyeron 142 por presentar subregistro de información en la tarjeta de registro del tratamiento antituberculoso. Esta tarjeta se utiliza para el proceso de validación de la condición de egreso del tratamiento y dar cumplimiento al objetivo 2; por lo tanto, se incluyeron 184 PPL (figura 2), cuyas características sociodemográficas y clínicas demuestran que 126 (68,4%) de los PPL con TB tienen entre 25 y 49 años, 144 (78,2%), proceden de la provincia del Guayas y, de estos, 44% provienen de los cuatros distritos de salud con incidencia de tuberculosis más alta (figura 3). Se identificaron 168 (91,3%) casos nuevos de TB pulmonar en el CPL-G1 varones, los cuales no tuvieron un tratamiento oportuno, ni se cumplió con el envío de realizar la prueba de sensibilidad a drogas (PSD) antes de iniciar el tratamiento (cuadro 2).

Elaboración propia con base en la información obtenida de la base de datos de la Estrategia de Prevención y Contol de Tuberuclosis, CPL-G1 varones.

**CUADRO 2.** Características sociodemográficas y clínicas de la población con tuberculosis en el CPL-G1 varones, Ecuador (2015-2016)

Características sociodemográficas y clínicas	n	%
Población total	184	100,0
Edad (años) (mediana, Q1-Q3)		
17-24	52	28,2
25-49	126	68,4
50-64	6	3,2
Peso (kg) (mediana, Q1-Q3)	50,4	46,5
Zona de origen		
1^a^	1	0,5
3^b^	2	1,0
4^c^	1	0,5
5^d^	32	17,3
7^e^	4	2,1
8^f^	144	78,2
Forma clínica		
Pulmonar	181	98,3
Extrapulmonar	3	1,6
Condición de ingreso		
Nuevo	168	91,3
Recaída	10	5,4
Pérdida en el seguimiento	6	3,2
Frotis de diagnóstico^g^		
Negativo	42	22,8
+	99	53,8
++	33	17,9
+++	10	5,4
Cultivo de diagnóstico		
Negativo	11	5,9
1-20 colonias	15	8,1
+	25	13,5
++	26	14,1
+++	10	5,4
Tiempo de inicio de tratamiento (días) (mediana, Q1-Q3)	8	5,1
≤ 3	37	20,1
≥ 3	147	78,8
Prueba de tamizaje de VIH	175	95,1
Coinfección TB/VIH	12	6,5
No reactivo	166	90,2
Sin prueba	6	3,2
Comorbilidad con diabetes	1	0,5

a Provincias de Esmeraldas, Imbabura, Carchi y Sucumbíos.

b Provincias de Cotopaxi, Tungurahua, Chimborazo y Pastaza.

c Provincias de Manabí y Santo Domingo de los Táschillas.

d Provincias de Santa Elena y Guayas (excepto cantón Guayaquil, Samborondón y Durán).

e Provincias de El Oro, Loja y Zamora Chinchípe.

f Cantón Guayaquil, Samborodón y Durán.

g El resultado del frotis de diagnóstico identifica el número de bacilos por microscopia; negativo: no se observan bacilos ácido-alcohol resistentes en 100 campos microscópicos; positivo +: < 1 bacilo/campo en promedio (de 10 a 99 bacilos) en 100 campos observados; positivo ++: 1-10 bacilos/campo en promedio en 50 campos observados; positivo +++: > 10 bacilos/campo en 20 campos observados.

Q1-Q3, cuartil 1-cuartil 3; VIH, virus de la inmunodeficiencia humana; TB, tuberculosis.

Elaboración propia,con base en la información obtenida de la base de datos de la Estrategia de Prevención y Control de Tuberculosis-CPL G1 varones.

Al analizar el desenlace del tratamiento en cada año, se observa que el porcentaje de fallecidos aumentó tres veces del 2015 al 2016 y el porcentaje de tratamiento exitoso se mantuvo por debajo del 90% en ambos años, (69,1% vs 71,6%) (figura 3).

Se identificó mayor riesgo de un tratamiento no exitoso, en el grupo de personas privadas de libertad (PPL) que presentan coinfección VIH con (RR: 1,66; IC95%: 1,33-2,07). En el grupo etario de 50 a 64 años (RR: 1,97; IC95%: 0,80-4,87; *P*: 0,29), en las PPL afectadas con tuberculosis extrapulmonar con (RR: 1,33; IC95%: 0,32-5,48; *P*: 0,87) y en los que presentan condición de ingreso con una historia de pérdida en el seguimiento con (RR: 1,74; IC95%: 0,64-4,67; *P*: 0,2) (cuadro 3) se observó la tendencia hacia mayor riesgo de tratamiento no exitoso; sin embargo, los resultados no alcanzaron a ser estadísticamente significativos.

## DISCUSIÓN

Este primer estudio sobre la tuberculosis en una de las prisiones de Ecuador, la cual presenta el mayor número de pabellones para la reclusión de gran cantidad de PPL de toda clase de sentencia, confirma la alta incidencia de TB en prisiones cuando se la compara con la población general. Revela, también, la deficiencia en la captación de sintomáticos respiratorios y en el cumplimiento de la meta de la OMS de tratamiento exitoso.

Los CPL constituyen uno de los problemas de salud pública más graves en las Américas debido a la elevada tasa de incidencia de tuberculosis que presentan en comparación con la población general, lo cual ya ha sido reportado en otros estudios (11, 12). En diferentes CPL de Colombia, la incidencia encontrada fue de 505/100 000 (16), ocho veces menor a la incidencia que presenta Ecuador en el CPL–G1 Varones, que es de 3 947/100 000 habitantes. Esta diferencia se puede explicar por la tasa de incidencia que presenta Colombia a nivel nacional de 26,3/100 000 en comparación con la de Ecuador que, para el 2017, fue de 34,5/100 000. La ausencia de políticas claras en el manejo de la tuberculosis en las prisiones podría ser otra explicación. Sin embargo, en países de ingresos medios o bajos, la tasa de incidencia en prisiones es de 1 942,8/100 000, aproximadamente cuatro veces más alta que la tasa que presenta Colombia sin alcanzar a la de Ecuador. La mayor cantidad de los prisioneros que se encuentran internados en el CPL-G1 varones provienen de los distritos de salud con más alta tasa de incidencia la cual oscila entre 77 a 96/100 000 (6, 17).

Alcanzar la meta de captación de los SR (4%) ayudaría a acortar la cadena de transmisión en los CPL de Ecuador. Esto permitiría la detección y diagnóstico tempranos para tratar oportunamente la enfermedad y, de esta manera, cumplir con la reducción de la tasa de incidencia (90%) hasta el 2035 y lograr la ejecución de los tres pilares de la *estrategia Fin a la TB* (18). A la inversa, en Ecuador, el CPL muestra una alta incidencia de TB. A pesar de ello, los SR identificados (1 547) no alcanzan el valor de los SR esperados (2 394), que es de 4% (13). Esto evidencia que no se está realizando búsqueda activa de SR y que 4% es una meta muy baja para los centros penitenciarios, debido a que son áreas de mayor congregación y hacinamiento. Estudios realizados en Etiopía y en Costa Marfil muestran lo inverso en las prisiones, lo cual puede suceder debido a que existe menos población en las cárceles de ambos países y realizan búsqueda activa e implementación de políticas en los CPL (19).

El porcentaje de los tratamientos exitosos (curados y completos) en este estudio es de 70,4%, similar a la tasa de tratamientos exitosos que presentan las cárceles en otros estudios (20), aun incumpliendo el 90% de la tasa de éxito de tratamiento de la estrategia *Fin a la TB*.

Elaboración propia con base en información obtenida de la base de datos de la Estrategia de Prevención y Control de Tuberculosis, CPL-G1 varones.

**CUADRO 3.** Factores asociados con el tratamiento antibuerculoso no exitoso en las personas privadas de libertad en el CPL-G1 varones, Ecuador (2015-2016)

Característica	Tratamiento no exitoso		Total	RRC (IC95%)	Valor *P*	RRA (IC95%)
	n	%				
Edad (años)						
17-24	16	30,8	52	1		1
25-49	35	27,8	126	0,90 (0,5-1,4)	0,68	0,91 (0,52-1,57 )
50-64	3	50,0	6	1,63 (0,6-4,0)	0,29	1,97 (0,80-4,87)
Forma clínica						
Pulmonar	53	29,3	181	1		1
Extrapulmonar	1	33,3	3	1,13 (0,22-5,73)	0,88	1,33 (0,32-5,48)
Condición de ingreso						
Recaída	2	20,0	10	0,68 (0,19-2,42)	0,55	0,86 (0,24-3,10)
Nuevo	49	29,2	168	1		1
Pérdida en el seguimiento	3	50,0	6	1,71 (0,74-3,94)	0,20	1,74 (0,64-4,67)
Tiempo de inicio de tratamiento (días)						
≤ 3	12	32,4	37	1		1
≥ 3	42	28,6	147	0,88 (0,51-1,49)	0,64	0,87 (0,49-1,52)
Coinfección TB/VIH						
No reactivo	42	25,3	166	1		1
Reactivo	6	50,0	12	1,7 (1,56-1,85)	< 0,001	1,66 (1,33-2,07)
Sin prueba	6	100,0	6	NC	NC	NC

RRC, riesgo relativo crudo; RRA, riesgo relativo ajustado; IC95%, intervalo de confianza de 95%; NC, no corresponde.

Elaboración propia con base en la información obtenida de la base de datos de la Estrategia de Prevención y Control de Tuberculosis, CPL-G1 varones.

Se observa un porcentaje elevado entre los perdidos en el seguimiento (12,5%) y los no evaluados (10,8%) debido a la falta de protocolos, políticas y un sistema de información que reporte la estancia y salida del PPL del centro penitenciario, situación similar que ocurre en las prisiones de África del Sur (21), lo que conlleva a un elevado riesgo de tratamientos no exitosos.

Los factores asociados a tratamientos no exitosos son: ser adulto mayor (50-64 años de edad) por la vulnerabilidad que le otorga su estado inmunitario (22, 23), seguido del grupo de 17-24 años; tener tuberculosis pulmonar, lo que eleva el riesgo de contagio dentro de los centros penitenciarios; presentar antecedentes de pérdida de seguimiento en su condición de ingreso, lo que ocasiona dificultad en la adherencia al tratamiento; y coinfección VIH, debido a la problemática clínica de ambas patologías y al tratamiento administrado, con mayor probabilidad de desarrollar reacciones adversas a los medicamentos lo que origina abandono del tratamiento. Estos resultados se corroboran con otros estudios en esta misma población realizados en la Región de las Américas (16, 24).

Si bien no hubo asociación con el tiempo de inicio al tratamiento, se observa que 80% de las PPL inició su tratamiento tres días o más después de haber sido diagnosticado. Este indicador revela que no se está cumpliendo con un diagnóstico y administración oportuna del tratamiento, lo cual podría explicarse por retraso en la atención a la PPL SR y en la recolección y transporte de la muestra, no contar con laboratorio de diagnóstico dentro del CPL y falta de *stock* de medicamentos antituberculosos en el sistema en general (tanto en los ministerios de salud como de justicia), como ha sido reportado por Souza et al. en Brasil (25).

Este fue un estudio operativo y, por lo tanto, sujeto a las limitaciones de este tipo de investigación. Por la naturaleza retrospectiva del estudio, la recopilación de la información dependió de la calidad de la base de datos del programa de tuberculosis y de las tarjetas de registro del tratamiento antituberculoso; un porcentaje importante de estas contenían información faltante y pacientes duplicados. Estas fueron causas de exclusión del análisis, como se describe en la sección sobre los resultados. Además, solo se pudo evaluar las variables que se recopilan habitualmente en los registros, por lo que no se incluyeron aquellas que se sabe están relacionadas con las variaciones en el resultado del tratamiento de la tuberculosis, como tener adicciones a drogas ilícitas y al tabaquismo, presentar falta de conversión en el frotis al segundo mes de tratamiento, estar en un régimen de retratamiento, poseer un nivel de instrucción básico, características clínicas individuales y malnutrición, entre otros.

## CONCLUSIONES

La incidencia de tuberculosis en el CPL-G1 varones es mayor que en la población general nacional. El objetivo de 90% de los tratamientos exitosos establecido por la OMS/OPS no se cumple y el principal factor asociado es la coinfección TB-VIH. En la actualidad, no se aplica de manera efectiva la *hacia el Fin de la TB* en todos los CPL de Ecuador (26).

Emerge la necesidad de coordinación y trabajo articulado entre los ministerios de salud y de justicia para la implementación de procesos y protocolos de salud que logren controlar el contagio de la tuberculosis dentro de los CPL y en la comunidad en general, con una atención integral en salud a la PPL mediante la implementación y aplicación de todos los pilares y componentes de la estrategia *Fin a la TB*.

## Contribución de los autores.

FC, DM y FP concibieron el estudio original, planificaron los experimentos, recolectaron y analizaron los datos, interpretaron los resultados, escribieron y revisaron el manuscrito. Todos los autores han leído y aprobado el manuscrito y han contribuido significativamente al trabajo.

## Agradecimientos.

Esta investigación se llevó a cabo mediante la Iniciativa de Capacitación Estructurada en Investigación Operativa (SORT IT, por sus siglas en inglés), una alianza mundial dirigida por el Programa Especial de Investigación y Capacitación de Enfermedades Tropicales de la Organización Mundial de la Salud (OMS/TDR) y el Departamento de Enfermedades Transmisibles y Determinantes Ambientales de la Salud de la Organización Panamericana de la Salud (OPS).

## Financiamiento.

Se obtuvo financiamiento de la oficina subregional Andina de la OPS. Los financiadores no desempeñaron ningún papel en el diseño del estudio, la recopilación y análisis de los datos, la decisión de publicar ni la elaboración del artículo.

## Declaración.

Las opiniones expresadas en este manuscrito son únicamente responsabilidad de los autores y no reflejan necesariamente los criterios ni la política de la *RPSP/PAJPH* o la Organización Panamericana de la Salud.

## References

[1] 1. Organización Mundial de la Salud. Global Tuberculosis Report 2019. Disponible en: https://apps.who.int/iris/bitstream/handle/10665/329368/9789241565714-eng.pdf?ua=1

[2] 2. Organización Panamericana de la Salud/Organización Mundial de la Salud (OPS/OMS). Tuberculosis en las Américas 2018. Washington D.C.: OPS; 2018. Disponible en: http://iris.paho.org/xmlui/bitstream/handle/123456789/49510/OPSCDE18036_spa?sequence=2&isAllowed=y

[3] 3. Valença MS, Scaini JLR, Abileira FS, Gonçalves CV, von Groll A, Silva PEA. Prevalence of tuberculosis in prisons: risk factors and molecular epidemiology. Int J Tuberc Lung Dis. 2015;19(10):1182-7. 10.5588/ijtld.15.0126 26459530

[4] 4. Ayala G, Garay J, Aragon M, Decroo T, Zachariah R. Trends in tuberculosis notification and treatment outcomes in prisons: a country-wide assessment in El Salvador from 2009–2014. Rev Panam Salud Publica. 2016;39(1):38-43. 27754539

[5] 5. Zambrano LI, Fuentes I, Rodas-Ortez H, Maldonado M, Lara B, Sierra M, et al. Tuberculosis in prisons: Honduras, Central America, 2007–2014. J Formos Med Assoc. 2017;116(7):565-6. 10.1016/j.jfma.2017.03.006 28390752

[6] 6. Dara M, Acosta CD, Melchers NVSV, Al-Darraji HAA, Chorgoliani D, Reyes H, et al. Tuberculosis control in prisons: current situation and research gaps. Int J Infect Dis. 2015;32:111-7. 10.1016/j.ijid.2014.12.029 25809766

[7] 7. Nyasulu P, Mogoere S, Umanah T, Setswe G. Determinants of pulmonary tuberculosis among inmates at Mangaung maximum correctional facility in Bloemfontein, South Africa. Tuberculosis Research and Treatment. 2015. doi: 10.1155/2019/4578329 PMC4381858 25866677

[8] 8. Gonçalves Lisbôa Pereira A, Caminha Escosteguy C, Brito Gonçalves J, Espínola Marques MRV, Medeiros C, Souza da Silva MC. Factors associated with death from tuberculosis and treatment default in a general hospital in the city of Rio de Janeiro, 2007 to 2014. Revista de Epidemiologia e Controle de Infeccão. 2018;8(3). Disponible en: https://online.unisc.br/seer/index.php/epidemiologia/article/viewFile/10675/7568

[9] 9. World Health Organization. Global tuberculosis report 2017. Geneva: WHO; 2017. Disponible en: https://www.who.int/tb/publications/global_report/gtbr2017_main_text.pdf

[10] 10. Organización Mundial de la Salud. El control de la tuberculosis en prisiones. Ginebra: OMS; 2000. Disponible en: http://apps.who.int/iris/bitstream/10665/67826/1/WHO_CDS_TB_2000.281_spa.pdf

[11] 11. Organización Panamericana de la Salud. VI Reunión Regional 2013. Disponible en: https://www.paho.org/hq/dmdocuments/2013/VI-Reunion-Regional-2013-Esp-1.pdf

[12] 12. Bourdillon PM, Gonçalves CCM, Pelissari DM, Arakaki-Sanchez D, Ko AI, Croda J, et al. Increase in tuberculosis cases among prisoners, Brazil, 2009–2014. Emerg Infect Dis. 2017;23(3):496-9. 10.3201/eid2303.161006 PMC5382752 28221118

[13] 13. Ministerio de Salud Pública del Ecuador. Manual de procedimientos para la prevención y control de la Tuberculosis 2017. Quito: Ministerio de Salud Pública; 2017. Disponible en: https://www.salud.gob.ec/wp-content/uploads/2017/07/MANUAL-DE-PROCEDIMIENTOS-DE-TB-FINAL.pdf

[14] 14. Ministerio de Salud Pública del Ecuador. Guia de práctica clínica de tuberculosis 2018. Disponible en: https://www.salud.gob.ec/wp-content/uploads/2018/03/GP_Tuberculosis-1.pdf.

[15] 15. World Health Organization. Definitions and reporting framework for tuberculosis – 2013 revision. Geneva: OMS; 2013. Disponible en: https://apps.who.int/iris/bitstream/handle/10665/79199/9789241505345_eng.pdf?sequence=1

[16] 16. Rueda ZV, López L, Vélez LA, Marín D, Giraldo MR, Pulido H, et al. High incidence of tuberculosis, low sensitivity of current diagnostic scheme and prolonged culture positivity in four Colombian prisons: a cohort study. PLoS ONE. 2013;8(11). Disponible en: https://www.ncbi.nlm.nih.gov/pmc/articles/PMC3836852/10.1371/journal.pone.0080592 PMC3836852 24278293

[17] 17. Dara M, Chadha SS, Melchers NV, van den Hombergh J, Gurbanova E, Al-Darraji H, et al. Time to act to prevent and control tuberculosis among inmates. Official Statement of The International Union Against Tuberculosis and Lung Disease. Int J Tuberc Lung Dis. 2013;17(1):4-5 Disponible en: https://www.ingenta​connect.com/content/iuatld/ijtld/2013/00000017/00000001/art00004%3bjsessionid=3ijlmhgunviq9.x-ic-live-03# 10.5588/ijtld.12.0909 23231999

[18] 18. Harries AD, Lin Y, Kumar AMV, Satyanarayana S, Takarinda KC, Dlodlo RA, et al. What can national TB control programmes in low- and middle-income countries do to end tuberculosis by 2030? F1000Research. 2018;7. Disponible en: https://www.ncbi.nlm.nih.gov/pmc/articles/PMC6039935/10.12688/f1000research.14821.1 PMC6039935 30026917

[19] 19. Séri B, Koffi A, Danel C, Ouassa T, Blehoué M-A, Ouattara E, et al. Prevalence of pulmonary tuberculosis among prison inmates: a cross-sectional survey at the correctional and detention facility of Abidjan, Côte d’Ivoire. PLoS ONE. 2017;12(7). Disponible en: https://www.ncbi.nlm.nih.gov/pmc/articles/PMC5536365/10.1371/journal.pone.0181995 PMC5536365 28759620

[20] 20. Adane K, Spigt M, Dinant G-J. Tuberculosis treatment outcome and predictors in northern Ethiopian prisons: a five-year retrospective analysis. BMC Pulm Med. 2018;18. Disponible en: https://www.ncbi.nlm.nih.gov/pmc/articles/PMC5819685/10.1186/s12890-018-0600-1 PMC5819685 29463234

[21] 21. Mnisi T, Tumbo J, Govender I. Factors associated with pulmonary tuberculosis outcomes among inmates in Potchefstroom Prison in North West province. South Afr J Epidemiol Infect. 2013;28(2):96-101.

[22] 22. Yew WW, Yoshiyama T, Leung CC, Chan DP. Epidemiological, clinical and mechanistic perspectives of tuberculosis in older people. Respirology. 2018;23(6):567-75. 10.1111/resp.13303 29607596

[23] 23. Byng-Maddick R, Noursadeghi M. Does tuberculosis threaten our ageing populations? BMC Infect Dis. 2016;16(1):119. 10.1186/s12879-016-1451-0 PMC4787032 26968654

[24] 24. Ribeiro Macedo L, Reis-Santos B, Riley LW, Maciel EL. Treatment outcomes of tuberculosis patients in Brazilian prisons: a polytomous regression analysis. Int J Tuberc Lung Dis 2013;17(11):1427-34. 10.5588/ijtld.12.0918 24125446

[25] 25. Souza KMJ de, Villa TCS, Assolini FEP, Beraldo AA, França U de M, Protti ST, et al. Delay in the diagnosis of tuberculosis in prisons: the experience of incarcerated patients. Texto Amp Contexto - Enferm. 2012;21(1):17-25.

[26] 26. Biadglegne F, Rodloff AC, Sack U. Review of the prevalence and drug resistance of tuberculosis in prisons: a hidden epidemic. Epidemiol Infect. 2015;143(5):887-900. 10.1017/S095026881400288X PMC9507155 25376279

[27] 27. Ministerio de Salud Pública de Ecuador. Manual de procedimientos para la prevención y control de la tuberculosis 2017. Quito: Ministerio de Salud Pública; 2017. Disponible en: https://www.salud.gob.ec/wp-content/uploads/2017/07/MANUAL-DE-PROCEDIMIENTOS-DE-TB-FINAL.pdf

